# A pilot study of microRNA assessment as a means to identify novel biomarkers of spontaneous osteoarthritis in dogs

**DOI:** 10.1038/s41598-022-22362-2

**Published:** 2022-10-28

**Authors:** Atsushi Yamazaki, Yuma Tomo, Hinano Eto, Koji Tanegashima, Kazuya Edamura

**Affiliations:** Laboratory of Veterinary Surgery, Department of Veterinary Medicine, College of Bioresource and Sciences, Nihon University, Fujisawa, Kanagawa Japan

**Keywords:** Biomarkers, Translational research, Animal physiology, Orthopaedics, Osteoarthritis

## Abstract

MicroRNAs (miRNAs) are important regulators of intercellular signaling and are promising biomarkers in osteoarthritis (OA). In this study, comprehensive analysis was performed to identify miRNAs involved in the pathogenesis of spontaneous OA in dogs. Dogs diagnosed with OA based on radiography and arthroscopy of the stifle joint were included in the OA group. Dogs without any evidence of orthopedic disease were included in the unaffected group. To investigate miRNA expression levels, RNA sequencing analysis (RNA-seq) was performed in synovial tissue (OA group: n = 3, Unaffected group: n = 3) and RT-qPCR was performed in synovial tissue, synovial fluid and serum (OA group: n = 17, Unaffected group: n = 6), and compared between the two groups. The RNA-seq results showed that 57 miRNAs were significantly upregulated and 42 were significantly downregulated in the OA group. Specifically, miR-542 and miR-543 expression levels in the synovial tissue, synovial fluid, and serum were consistently higher in the OA group than in the unaffected group, suggesting that these miRNAs may be used as biomarkers for detecting canine OA. This is the first report to comprehensively analyze the expression patterns of miRNAs in the synovial tissue of dogs with spontaneous OA.

## Introduction

Osteoarthritis (OA) is a disorder of synovial joints characterized by aberrant repair, eventual degeneration of articular cartilage, formation of new bone at the articular margins, sclerosis of the subchondral bone, and development of synovial inflammation^1^. Many studies have been conducted on the pathogenesis of OA in humans^2^. Recently, microRNAs (miRNAs) with 21–25 nucleotides have been identified as novel regulators of cartilage homeostasis and OA pathogenesis and are considered promising diagnostic and therapeutic biomarkers^3^ for detecting OA. miRNAs are important cellular regulators that target mRNAs and regulate gene expression under various physiological and disease conditions^4^. It has been reported that synovial fibroblast-derived exosomal miRNA-126-3p was sufficient to suppress the formation of osteophytes, prevent cartilage degeneration, and exert anti-apoptotic and anti-inflammatory effects on articular cartilage in rat OA model^5^. miRNAs are secreted into the extracellular space via exosomes, which are extracellular vesicles, and play an important role in cellular communication^6^. Thus, extracellularly secreted miRNAs can be used as a measure of both the environment and cellular state^6^.

A review investigating the role of miRNAs in human OA showed that miRNA expression patterns are altered in degenerated articular cartilage, affecting articular cartilage homeostasis^7^. Another review reported that miRNA expression patterns in synovial tissue were also altered in OA, affecting the regulatory mechanisms of synovial tissue metabolism and inflammation^8^. Furthermore, comprehensive analyses of miRNA expression in the blood of patients with OA revealed that several miRNAs were significantly up- or downregulated in the blood when compared to that of people without OA. miRNAs thus identified, could serve as biomarkers for detecting OA^9,10^.

Canine OA has become more prevalent as the lifespan of dogs increases with the advancement of veterinary diagnostic and therapeutic techniques and technologies. Therefore, OA has become a major concern in veterinary medicine. One report estimated that approximately 20% of adult dogs in the United States are affected by OA^11^. Although OA is one of the major causes of chronic pain in dogs, its pathogenesis is not fully understood. Most studies on OA pathogenesis in dogs have focused on the expression profile of cytokines and pro-inflammatory mediators in synovial tissue and articular cartilage^12–14^. In OA pathogenesis, cartilage breakdown products released into the synovial fluid are phagocytosed by synovial cells, causing synovial inflammation^15^. In this situation, synovial cells produce excessive amounts of inflammatory cytokines and proteolytic enzymes responsible for cartilage breakdown, further progressing OA pathogenesis^15^. Since synovitis plays an important role in the pathogenesis of OA, analysis of miRNAs involved in the regulation of synovitis will help understand the pathogenesis of OA. If miRNAs with altered expression in the synovial fluid or blood of dogs with spontaneous OA can be detected, they may be utilized as biomarkers for canine OA. To the best of our knowledge, no studies have examined the expression levels and patterns of miRNAs or evaluated their potential as biomarkers for detecting spontaneous OA in dogs.

The objective of this study was to comprehensively analyze miRNA expression in the synovial tissue and identify those involved in the pathology of canine OA in dogs with spontaneous OA using RNA sequencing. miRNAs that were significantly up- or downregulated in the synovial tissue were identified, and candidate miRNAs that can be used as biomarkers for early diagnosis of canine OA were chosen. Furthermore, the expression levels of these miRNAs were measured in the synovial fluid and serum obtained from dogs with spontaneous OA using quantitative reverse transcription-polymerase chain reaction (RT-qPCR) to assess their usefulness as biomarkers for diagnosing canine OA.

## Results

### Case selection

For the small RNA-seq analysis, three dogs met the inclusion criteria of the OA group. The mean age and body weight of the dogs in the OA group were 6.9 ± 7.1 years (range 0.5 to 14.5 years) and 21.8 ± 5.3 kg (range 16.5 to 27.0 kg), respectively. Two neutered males and one intact female were included in the study. The breeds included were Akita, Border Collie, and Labrador Retriever. Two stifle joints had OA due to the rupture of the cranial cruciate ligament, and the remaining joint had OA due to lateral patellar luxation. In all the dogs in the OA group, severe synovitis was observed on arthroscopy. Clinical information of the OA group was listed in Table 1. Three healthy Beagle dogs without orthopedic diseases were included in the unaffected group. The mean age and body weight of the dogs in the unaffected group were 8.2 ± 5.3 years (range 2.1 to 11.3 years) and 11.1 ± 1.3 kg (range 10.0 to 12.5 kg), respectively. Two males and one female were included in the unaffected group.

**Table 1:** Clinical information in the OA group.

									Analysis	
Sample No	Breed	Gender	Age (y)	Bodyweight (kg)	BCS	Synovitis score	Cartilage score	OA score	RNA-seq	RT-qPCR
1	Border Collie	Neutered male	5.6	16.5	3/5	3/5	0/5	3/5	○	
2	Akita	Female	0.5	22.0	3/5	4/5	0/5	2/5	○	
3	Labrador Retriever	Neutered male	14.5	27.0	4/5	2/5	4/5	2/5	○	○
4	Mix	Neutered female	9.9	34.5	3/5	4/5	1/5	3/5		○
5	Mix	Neutered female	6.3	19.0	3/5	4/5	2/5	5/5		○
6	Golden Retriever	Neutered female	6.4	37.0	4/5	4/5	2/5	3/5		○
7	Labrador Retriever	Neutered female	6.7	25.8	4/5	4/5	2/5	4/5		○
8	Bernese Mountain Dog	Male	0.4	28.5	3/5	4/5	3/5	2/5		○
9	Toy Poodle	Neutered male	6.7	6.0	3/5	3/5	0/5	2/5		○
10	Jindo Dog	Neutered male	7.9	24.9	4/5	4/5	4/5	2/5		○
11	Bernese Mountain Dog	Neutered female	7.8	36.5	5/5	2/5	4/5	2/5		○
12	Welsh Corgi Pembroke	Neutered female	4.6	13.3	3/5	4/5	0/5	4/5		○
13	American Cocker Spaniel	Neutered female	7.5	11.3	3/5	3/5	4/5	3/5		○
14	Jack Russell Terrier	Neutered female	5.4	6.3	3/5	4/5	1/5	4/5		○
15	Border Collie	Neutered female	9.1	18.5	3/5	3/5	0/5	3/5		○
16	Labrador Retriever	Neutered male	8.1	43.8	5/5	4/5	4/5	1/5		○
17	Siberian Husky	Neutered male	11.9	24.5	3/5	2/5	2/5	2/5		○
18	Golden Retriever	Neutered female	2.1	25.6	3/5	4/5	0/5	1/5		○
19	Siberian Husky	Neutered male	8.2	27.8	3/5	4/5	2/5	2/5		○

Seventeen dogs met the inclusion criteria of the OA group for the RT-qPCR analysis. The mean age and body weight of dogs in the OA group were 7.3 ± 3.3 years (range 0.4 to 14.5 years) and 24.1 ± 10.7 kg (range 6.3 to 43.8 kg), respectively. This group included seven males (one intact and six neutered) and ten spayed females. The breeds included were Labrador Retriever (n = 3), Bernese Mountain Dog (n = 2), Golden Retriever (n = 2), Siberian Husky (n = 2), Mix (n = 2), American Cocker Spaniel (n = 1), Border Collie (n = 1), Jack Russell Terrier (n = 1), Jindo Dog (n = 1), Toy Poodle (n = 1), and Welsh Corgi Pembroke (n = 1). Sixteen stifle joints had OA due to rupture of the cranial cruciate ligament, and one had OA due to lateral patellar luxation. Moderate or severe synovitis was observed on arthroscopy in all animals in the OA group. Detailed clinical information of the OA group was shown in Table 1. In contrast, six healthy Beagle dogs without orthopedic diseases were included in the unaffected group. The mean age and body weight of the unaffected group were 2.1 ± 0.3 years (range 2.0 to 2.7 years) and 10.2 ± 1.8 kg (range 6.7 to 11.6 kg), respectively. Four intact males and two intact females were included in the unaffected group.

### Small RNA-sequencing

**Figure 1:**
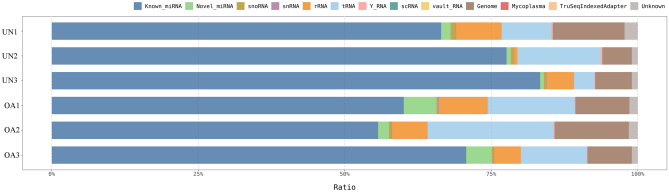
Summary of small RNA composition. UN, unaffected group; OA, osteoarthritis group.

When the quality of the data obtained by RNA sequencing of library samples was assessed, the average Q30 was 96.8 ± 0.3%, indicating that the quality of RNA sequencing was very high (Supplementary Table 1). The average count after trimming the adapter and rRNA sequences was 18,778,784 ± 2,919,189 reads, which was sufficient for comprehensive miRNA analysis (Supplementary Table 2). Furthermore, when the RNA composition was investigated, approximately 60% of the detected RNA were known miRNAs in each sample, and the amount of miRNA was sufficient for analysis (Fig. 1).

**Figure 2:**
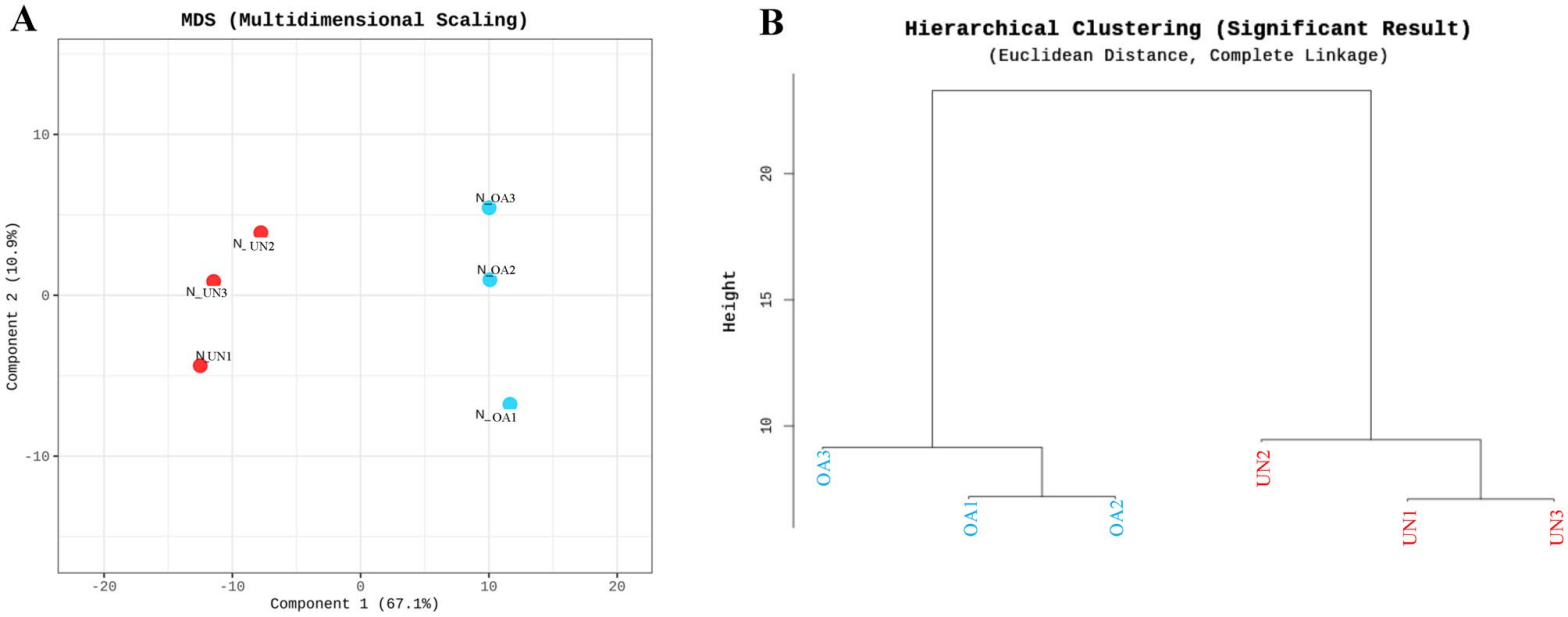
The results of multi-dimensional scaling and hierarchical clustering. Using each sample's normalized value, the similarity between samples was graphically shown in a 2D plot to show the variability of the total data (**A**), and the high expression similarities were grouped (**B**) (Distance metric = Euclidean distance, Linkage method = Complete linkage). The composition of miRNAs in samples from the OA and unaffected groups was similar in each group. UN, unaffected group; OA, osteoarthritis group.

miRNAs with zeroed counts across more than 51% of all samples were excluded during pre-processing, and 281 mature miRNAs were analyzed. When the multi-dimensional scaling (MDS) method and hierarchical clustering analysis were performed, the miRNA composition in the samples of the two groups was similar between the OA and unaffected groups (Fig. 2).

**Figure 3:**
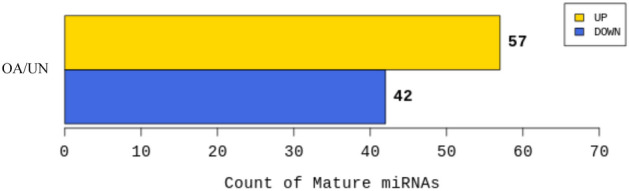
Up- and down-regulated count of miRNAs by fold change and *p*-value. Shows number of up- and down-regulated mature miRNAs based on fold change and *p*-value of comparison pair. The results showed that 57 miRNAs were significantly upregulated and 42 were significantly downregulated in the OA group. UN, unaffected group; OA, osteoarthritis group.

**Figure 4:**
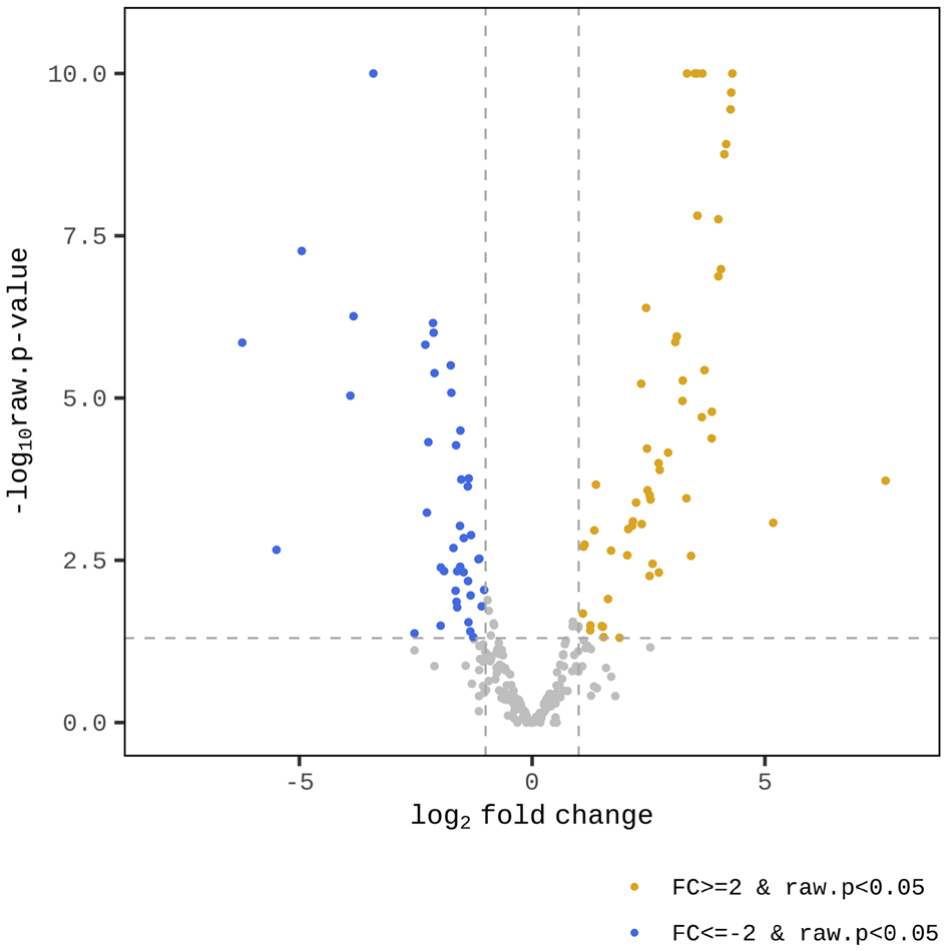
Volcano plot of the expression level of two groups. Log_2_ fold change and *p*-value obtained from the comparison between two groups plotted as a volcano plot (X-axis, log_2_ fold change; Y-axis, -log_10_ *p*-value).

**Figure 5:**
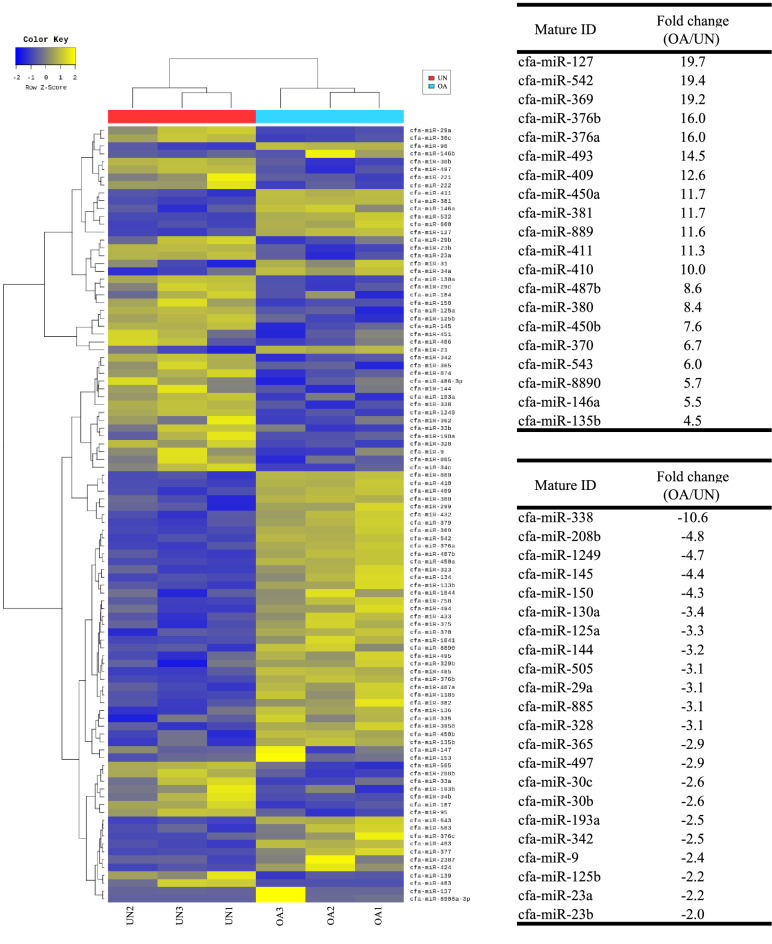
Heat map of the one-way hierarchical clustering using Z-score for normalized value. (**A**) Heatmap shows the result of hierarchical clustering analysis of mature miRNAs and samples by expression level (normalized value) from a significant list. (**B**) miRNAs with low sample-to-sample variability in each group. UN, unaffected group; OA, osteoarthritis group.

The 57 and 42 miRNAs were significantly up- and downregulated in the OA group, respectively (Fig. 3). In addition, a volcano plot was created based on the log_2_ fold change and *p*-value calculated for comparison of miRNA expression levels between the OA and the unaffected groups, which revealed that the expression level was obviously different between the two groups (Fig. 4). A heat map was created by hierarchical clustering analysis to select miRNAs with low sample-to-sample variability in each group, which showed that 20 miRNAs were significantly upregulated and 22 miRNAs were significantly downregulated in the OA group (Fig. 5).

### RT-qPCR for supporting

To support the results of small RNA-seq by RT-qPCR, miRNAs with large fold changes in expression levels or miRNAs that have been reported to be expressed in OA-affected knee joints in humans^7,9^ were selected from the 20 miRNAs that were significantly upregulated in the OA group as novel biomarker candidates. The selected miRNAs included miR-543, miR-146a, miR-127, miR-381, miR-542, and miR-369, and their expression levels in synovial tissue, synovial fluid, and serum were examined using RT-qPCR. Samples in which housekeeping genes were not detected were excluded from the study.

**Figure 6:**
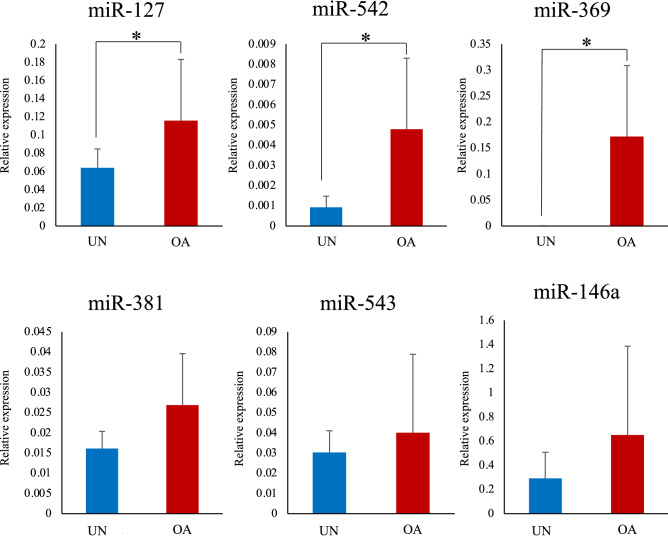
Comparison of miRNA expression levels in the synovial tissue between the two groups. The relative expression levels of each miRNA in the synovium in the osteoarthritis (OA) group and the unaffected group were compared. The data are presented as mean ± standard deviation. UN, unaffected group; OA, osteoarthritis group. *: Mean values differ significantly between the groups (*p* < 0.05).

The expression levels of miR-127, miR-542, and miR-369 in the synovial tissues of the OA group were significantly higher than those in the unaffected group (Fig. 6). The expression levels of miR-381, miR-543, and miR-146a were higher in the OA group than in the unaffected group (Fig. 6). These results were similar to those of the small RNA-seq. Furthermore, the expression level of miR-542 showed significant positive correlations with synovitis score and radiographic OA score. The expression levels of miR-127 and miR-369 were significantly positively correlated only in the radiographic OA score.

**Figure 7:**
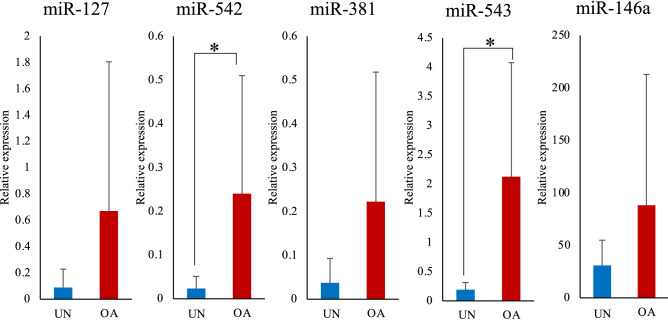
Comparison of miRNA expression level in the synovial fluid between the two groups. The relative expression levels of each miRNA in the synovial fluid in the osteoarthritis (OA) group and the unaffected group were compared. The data are presented as mean ± standard deviation. UN, unaffected group; OA, osteoarthritis group. *: Mean values differ significantly between the groups (*p* < 0.05).

The expression levels of miR-542 and miR-543 were significantly higher in the synovial fluid of the OA group than in those of the unaffected group (Fig. 7). The expression level of miR-542 showed significant positive correlations with synovitis score and radiographic OA score, and the expression levels of miR-543 were significantly positively correlated only in the radiographic OA score. In addition, miR-127, miR-381, and miR-146a expression levels tended to be higher than those in the unaffected group (Fig. 7). However, miR-369 was not detected in synovial fluid. Therefore, we investigated the expression levels of miR-127, miR-542, miR-381, miR-543, and miR-146a in serum samples.

**Figure 8:**
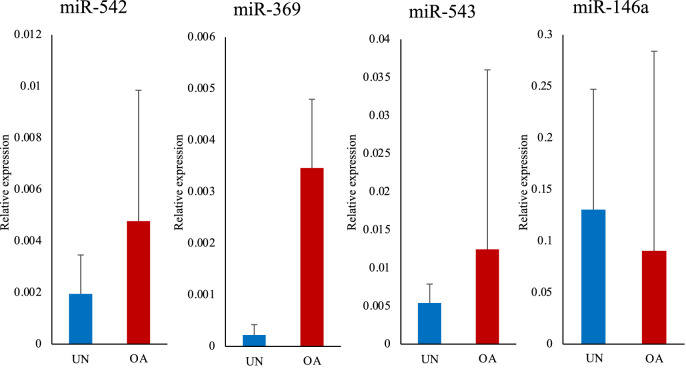
Comparison of miRNA expression level in the serum between the two groups. The relative expression levels of each miRNA in the serum in the osteoarthritis (OA) group and the unaffected group were compared. The data are presented as mean ± standard deviation. UN, unaffected group; OA, osteoarthritis group. *: Mean values differ significantly between the groups (*p* < 0.05).

In the serum of the OA group, miR-542 and miR-543 expression levels were higher than those of the control group. In contrast, the miR-146a expression level was slightly lower than that in the unaffected group (Fig. 8). miR-127 was not detected in serum (Fig. 8). There was no correlation between miRNA expression levels of serum and both synovitis score and radiographic OA score.

## Discussion

This study demonstrated that miR-542 and miR-543 expression levels were consistently higher in synovial tissue, synovial fluid, and serum of dogs with spontaneous OA.

Using small RNA-seq, it was showed that the miRNA composition in the OA group was specific and completely different from that of the unaffected group. Specifically, we found that 52 miRNAs were significantly upregulated and 44 were significantly downregulated in the synovial tissue of the OA group. These results suggest that the expression of miRNAs is markedly altered in OA affected joints in dogs. After excluding miRNAs with large differences in expression levels among samples in each group using the heat map (Fig. 5), 20 and 22 miRNAs with significantly high and low expression levels, respectively, were selected as miRNAs that may be involved in canine OA pathogenesis. Furthermore, among the miRNAs with significantly high expression levels, miRNAs with large fold changes in expression levels or miRNAs that were altered in articular cartilage and synovial tissues of human OA were identified as candidates for novel biomarkers. When these miRNA expression levels were quantified by RT-qPCR, the results were similar to those obtained using small RNA-seq. Therefore, miRNAs with consistent results in the two examinations, including miR-543, miR-146a, miR-127, miR-381, miR-542, and miR-369, may play a significant role in the pathology of spontaneous OA in dogs.

In humans, miRNA expression levels are altered in the synovial tissue of OA patients^8,16^. Among these miRNAs, only miR-146a was expressed in the synovial tissues of dogs with spontaneous OA in the present study. miR-146a has been investigated in many institutions as a miRNA associated with the pathogenesis of human OA^8^. In a previous study investigating the miR-146a expression level in cultivated synovial fibroblasts of humans, the expression level was higher than that in normal synovial fibroblasts^17^. Another study examining the miR-146a expression level in the articular cartilage of human OA patients showed a significantly higher expression level than that in normal articular cartilage^18^. Furthermore, it has been reported that the miR-146a expression level in the plasma of human OA patients was significantly higher than that in the plasma of control subjects^9^. Thus, miR-146a may be involved in the pathogenesis of OA in humans. It has been shown that canine miR-146a has the same base sequence as human miR-146a or miR-146a-5p^19^. In addition, the results of the present study revealed that the miR-146a expression level was significantly higher in the synovial tissue of spontaneous canine OA than that in unaffected dogs. Therefore, miR-146a may be involved in the pathogenesis of canine OA.

In the present study, miR-127, miR-542, and miR-369 expression levels in the synovial tissue of canine OA stifle joints were significantly higher, and the miR-381 and 543 expression levels were higher than those in unaffected dogs. It has been shown that the base sequence of canine miR-127, miR-542, miR-369, miR-381, and miR-543 are the same as those of human miR-127-3p, miR-542-3p, miR-369-3p, miR-381-3p, and miR-543, respectively^19^. Although the relationship between inflammatory diseases and miR-381-3p and miR-543 has not been clarified, it has been reported that miR-127-3p was involved in inflammatory bowel disease in humans^20^. In addition, in vitro studies using an inflammation model in murine and human cells revealed that miR-542-3p and miR-369-3p are involved in regulating the inflammatory response^21,22^. In dogs, studies on the expression levels of these miRNAs have been performed mainly in tumors^23,24^, but their role in inflammatory tissues is unknown. In the present study, miR-127, miR-542, miR-369, miR-381, and miR-543 were expressed at high levels in the synovial tissues of spontaneous canine OA, suggesting that these miRNAs may be involved in the regulation of synovitis in canine OA. Further studies are needed to reveal the physiological roles of these miRNAs in synovitis in dogs with spontaneous OA.

miR-542 and miR-543 expression levels in the synovial fluid of dogs with spontaneous OA were significantly higher than those in unaffected dogs. Furthermore, the study revealed that the expression levels of miR-542 and miR-543 in synovial tissue and synovial fluid were significantly positively correlated with severity of synovitis or radiographic OA score. It has been reported that miRNAs in the synovial fluid are derived from synovial tissue, and the miRNAs in synovial fluid are stable^25^. Therefore, the significantly higher expression levels of miR-542 and miR-543 in the synovial fluid might reflect the state of synovial tissue in dogs with spontaneous OA.

In this study, the miRNAs that were consistently expressed at higher levels in the synovial tissue, synovial fluid, and serum of dogs with spontaneous OA were miR-542 and miR-543. In humans, there is limited information on circulating miRNAs in patients with OA. In a previous study investigating the expression levels of circulating miRNAs in the plasma of human OA patients, seven miRNAs, including miR-543, were found to be significantly higher in the early stage than in the late stage of the disease^10^. In addition, it has also been reported that circulating miRNAs may reflect the early signal that initiates a cascade of events that contribute to OA^10^. To the best of our knowledge, circulating miRNAs have not yet been investigated in dogs with spontaneous OA. In the present study, miR-542 and miR-543 expression levels were significantly higher not only in the serum but also in the synovial tissue or synovial fluid of dogs affected by OA than in those of unaffected dogs, suggesting that these miRNAs may be useful as biomarkers for canine OA. However, further studies are needed to demonstrate that miR-542 and miR-543 are specific biomarkers for canine OA.

In this study, the genes targeted by miR-542 and 543 and their associated pathways were also investigated. However, due to the lack of pathway information related to the pathogenesis of OA in the canine database, the pathways associated with the pathogenesis of OA involving these miRNAs were unable to be identified. Therefore, further studies are needed to clarify the pathways in canine OA pathology involving miR-542 and miR-543 target genes.

Since the identification of OA subtypes is important for the development of treatments targeting specific types of OA pathology, analysis of the expression patterns of genes associated with OA conditions has been actively conducted in recent years to identify subtypes of human OA^26^. However, the pathogenesis of canine OA is still largely unexplored, and no studies have been conducted to identify subtypes of canine OA. In this study, several miRNAs associated with canine OA were identified, but further analysis is needed to identify subtypes of canine OA based on miRNAs.

One of the key limitations of this study is that we only examined miRNA expression in a small number of dogs. In addition, age and breed bias may affect the results of miRNA measurements. In order to identify miRNAs involved in the pathogenesis of OA, it is necessary to analyze novel miRNAs and non-coding RNAs, and miRNAs expressed in articular cartilage as well as synovial tissue. Therefore, additional studies should be conducted with a larger sample size. Since NSAIDs with anti-inflammatory properties were administered in this study, it is undeniable that this drug affects the expression of miRNAs involved in inflammation. The expression of miRNAs in dogs with other diseases was not included in this study. In addition, all miRNAs that showed significant changes on RNA-Seq were not examined. Further studies are needed to identify the best candidate miRNAs for the diagnosis of spontaneous OA in dogs.

In conclusion, the miRNAs expressions in the synovial tissue of the dogs with spontaneous OA were comprehensively analyzed, and their expression pattern was clarified. In addition, the present study demonstrated that miR-543, miR-146a, miR-127, miR-381, miR-542, and miR-369 might be involved in the pathogenesis of spontaneous OA in dogs. Furthermore, miR-542 and miR-543 expression levels tended to be consistently high in all samples, suggesting that these miRNAs may be useful biomarkers for detecting canine OA. This study is expected to contribute to the elucidation of the pathogenesis of canine OA and the development of new diagnostic methods and drugs for spontaneous OA in dogs.

## Materials and methods

### Case selection

Dogs that presented to the Animal Medical Center of Nihon University between October 2019 and October 2020 and were diagnosed with stifle OA based on radiographic and arthroscopic findings were included in the OA group. First, breed, age, gender, body weight, and body condition score (BCS) were recorded. In addition, the severity of synovitis, the degree of articular cartilage damage (modified Outerbridge scale), and radiographic OA score were evaluated based on previous reports^27–29^. This was a prospective study and the dogs included in the study were randomly selected. Dogs with suspected or documented neurological or immune-mediated diseases were excluded from the OA group. This study on owner-owned dogs was approved by the Clinical Research and Trial Ethics Committee of the Animal Medical Center of Nihon University (ANMEC-3-002), and consent was obtained from all owners of the dogs to use the samples collected for this study.

Dogs with no evidence of orthopedic disease were assigned to the unaffected group. This study on healthy laboratory dogs was approved by the Nihon University Animal Use and Care Committee (AP19BRS066-1).

All experiments were conducted in accordance with relevant guidelines and regulations and complies with the ARRIVE guidelines.

### Samples

In the OA group, all samples were collected at the time of surgery on the affected stifle joint under general anesthesia. The dogs were premedicated with atropine sulfate (0.04 mg/kg; Nipro ES Pharma, Osaka, Japan) subcutaneously and fentanyl citrate (5 μg/kg; Daiichi Sankyo Propharma, Tokyo, Japan) intravenously. Subsequently, anesthesia was induced by administering propofol (4.0 mg/kg; Zoetis Japan, Tokyo, Japan) intravenously. After intubation, anesthesia was maintained with isoflurane (1.5 to 2.0%; DS Pharma Animal Health, Osaka, Japan) in 100% oxygen given in an endotracheal tube. Perioperative analgesia was provided by administrating meloxicam (0.1 mg/kg; Boehringer Ingelheim Animal Health Japan, Tokyo, Japan) subcutaneously and fentanyl citrate (5 to 10 μg/kg/h, constant rate infusion) intravenously.

Synovitis of the stifle joint affected by OA was evaluated by stifle arthroscopy based on previous report^27^, and synovial tissue samples were collected from the evaluated sites. Collected synovial tissue samples were immediately frozen in liquid nitrogen. Synovial fluid and serum samples were also collected from the same dogs. Synovial fluid was collected from the stifle joint affected by OA and centrifuged at 1000×*g* for 20 min. The supernatant of the centrifuged synovial fluid was then collected. Blood was collected from the external jugular vein into serum separator tubes and placed on the benchtop at room temperature for 30 min. The blood was then centrifuged at 12,000×*g* for 90 s to obtain the serum samples. All collected samples were stored in a deep freezer at − 80 °C until RNA extraction.

In the unaffected group, synovial tissue samples were collected from the stifle joints under general anesthesia after confirming that there was no evidence of synovitis^27^. These subject dogs were premedicated with butorphanol tartrate (0.2 mg/kg; Meiji Seika Pharma, Tokyo, Japan) and medetomidine hydrochloride (20 μg/kg; Kyoritsu Seiyaku, Tokyo, Japan) intramuscularly. Subsequently, anesthesia was induced by administering 4.0 mg/kg propofol intravenously and was maintained with 1.5% to 2.0% isoflurane in 100% oxygen given in an endotracheal tube. These dogs were received appropriate post-operative management including administration of analgesics and antibiotics according to institutional regulations. Synovial fluid and serum were collected from the same dogs, and all collected samples were stored as described above.

### Total RNA extraction

Cryopreserved synovial tissue samples were crushed in liquid nitrogen, immediately dissolved in 1 ml of TRIzol reagent (Thermo Fisher Scientific Inc., Waltham, MA, USA), and homogenized. Subsequently, 200 μl of chloroform was added to the homogenized tissue solution and centrifuged at 12,000×*g* for 15 min at 4 °C to obtain an aqueous layer containing RNA. Total RNA was extracted using the miRNeasy Mini Kit (QIAGEN, Hilden, Germany), and DNase treatment was performed using the RNase-Free DNase set (QIAGEN). Finally, total RNA was eluted in 30 μl of RNase-free water.

Cryopreserved synovial fluid and serum samples were thawed at room temperature and centrifuged at 16,000×*g* for 5 min. Next, 750 μl of TRIzol LS reagent (Thermo Fisher Scientific Inc.) was added to 250 μl of the supernatant collected from each sample, and the solutions were mixed by vortexing for 15 s. Next, 200 μl of chloroform was added to the mixed solution and centrifuged at 12,000×*g* for 15 min at 4 °C to obtain an aqueous layer containing RNA. Total RNA was extracted using the miRNeasy Serum/Plasma kit (Qiagen) and eluted in 14 μl of RNase-free water. The total RNA concentration and the 260/280 ratio of synovial tissue, synovial fluid, and serum samples were measured using NanoDrop One (Thermo Fisher Scientific Inc.).

### Small RNA sequencing

The TruSeq Small RNA Library Prep Kit (Illumina, Inc., CA, USA) was used to prepare the small RNA library. The adenylated single-strand DNA 3' adapter, followed by a 5' adapter, were ligated to the small RNAs. The adapters were designed to capture small RNAs with 5' phosphate groups, which are characteristic of miRNAs, rather than RNA degradation products with a 5' hydroxyl group. miRNA fragments with ligated adapters were converted to cDNA fragments, which were later used in the sequencing reaction. PCR was performed to amplify the cDNA sequence pool. The amplified cDNA fragments were electrophoresed on an agarose gel, and the bands containing the molecules corresponding to the miRNA fragments with ligated adapters were cut out for subsequent sequencing (library size ranged from 145 to 160 bp). The cDNA fragments were sequenced using HiSeq 2500 (Illumina, Inc.), and approximately 1 billion single-end reads were sequenced per sample with a 51-bp read length.

### Sequencing data analysis

Raw reads of small RNAs were pre-processed to eliminate adapter sequences. If a sequence was matched to more than the first 5 bp of the 3' adapter sequence, it was regarded as an adapter sequence and then trimmed from the read using Cutadapt (National Bioinformatics Infrastructure Sweden; Uppsala, Sweden). Trimmed reads longer than 18 bp were selected for mapping reliability. The remaining reads were classified as non-adapter reads, and their adapter sequences were not sequenced. Trimmed reads were used for downstream analyses. To eliminate rRNA, reads that were aligned with the 45S pre-rRNA and mitochondrial rRNA of *Canis lupus familiaris* were excluded.

Sequence alignment and detection of known miRNAs were performed using miRDeep2 (Max Delbrück Center for Molecular Medicine, Helmholtz Association, Berlin, Germany). rRNA-filtered reads were aligned to the mature and precursor miRNAs of *Canis lupus familiaris* obtained from miRBase v22.1^30^ using the miRDeep2 quantifier module. In addition, uniquely clustered reads were sequentially aligned to the reference genome, miRBase v22.1, and RNAcentral v14.0^31^ to identify known miRNAs and other types of RNA for classification.

### Differentially expressed miRNA analysis

The number of reads for each miRNA was normalized by the trimmed mean of M-values (TMM) normalization method using the edgeR package (Lucent Technologies, New Jersey, USA). miRNAs with zeroed counts across more than 51% of all samples were excluded in a pre-processing stage. The normalized read count of the filtered miRNAs was added to 1, and a log_2_ transformation was performed. For each miRNA, log counts per million (CPM) and log fold changes were calculated to compare the differences between the OA group and the unaffected group. Additionally, the MDS method was used to visualize the similarities among the samples, and the Euclidean distance was applied as a measure of dissimilarity. Hierarchical clustering analysis was also performed using complete linkage and Euclidean distance as a measure of similarity to display the expression patterns of differentially expressed miRNAs that had a |fold change|≥ 2 and *p* < 0.05. All data analyses and visualization of differentially expressed miRNAs were conducted using R (version 3.6.1; www.r-project.org).

### RT-qPCR for supporting

Reverse transcription was performed using miScript II RT Kit (QIAGEN) and My Genie 32 Thermal Block (BIONEER Co., Daejon, Korea). Briefly, 75 ng of total RNA was added to the reverse transcription master mix. Subsequently, reverse transcription was performed at 37 °C for 60 min, and the reverse transcriptase was inactivated by incubation at 95 °C for 5 s. The cDNA samples were stored in a deep freezer at -80℃ until RT-qPCR was performed.

RT-qPCR was performed using the miScript SYBR Green PCR kit (QIAGEN) and Thermal Cycler Dice Real Time System II (TaKaRa Bio Inc., Kusatsu, Shiga, Japan). The PCR reaction solution per well contained 12.5 μl of QuantiTect SYBR Green PCR Master Mix, 2.5 μl of miScript Universal Primer, 2.5 μl of miScript Primer Assay, 1 µl (template: 1.25 ng) of the first-strand cDNA, and 6.5 µl of RNase free water. Primers used in this study are listed in Supplementary Table 3. *RNU6-2* was used for the synovial tissue and the synovial fluid^4,32^, and *let-7a* was used for the serum^33^ as endogenous controls. First, the DNA polymerase was activated at 95 °C for 15 min. Each PCR cycle involved 40 cycles of denaturation at 94 °C for 15 s, annealing at 55 °C for 30 s, and extension at 70 °C for 30 s. The results were analyzed using the crossing point method and the comparative cycle threshold (ΔCt) method^34^ using TP900 DiceRealTime v4.02B (TaKaRa Bio, Inc.). RT-qPCR of no-template controls was performed with 1 μl RNase- and DNA-free water, and the specificity of the amplified PCR products was verified by melting curve analysis. Finally, the relative expression levels of each miRNA were compared between the two groups for each sample. Furthermore, the correlation between the relative expression level of each miRNA and synovitis score and radiographic OA score was also investigated.

### Statistical analysis

In the differentially expressed miRNA analysis, a statistical hypothesis test for comparing the two groups was conducted using the exact test in edgeR. miRNAs were considered to be significantly differentially expressed between two groups only if the false discovery rate *p*-value was < 0.05 and the absolute log2 of fold change was ≥ 2.0. In RT-qPCR analysis, the obtained data were calculated as the mean ± standard deviation. Statistical analyses were performed using GraphPad Prism version 6.0 for Macintosh (GraphPad Software Inc., San Diego, CA, USA). The Mann–Whitney test was used to compare the two groups. Spearman’s rank correlation coefficients were calculated to assess the correlation between the relative expression level of each miRNA and synovitis score and radiographic OA score. Statistical significance was set at *p* < 0.05.

## Supplementary Information

Supplementary Information.

## Data Availability

All data generated or analyzed during this study are included in this published article (and its Supplementary Information files). The datasets of RNA-seq generated and analyzed during the current study are available in the GEO data repository (https://www.ncbi.nlm.nih.gov/geo/; accession number GSE 212544).
