# Supplementary material for: A pilot study of microRNA assessment as a means to identify novel biomarkers of spontaneous osteoarthritis in dogs
Source: Sci Rep. 2022 Oct 28;12:18152. doi: 10.1038/s41598-022-22362-2 (PMC9616959; doi:10.1038/s41598-022-22362-2)
Supplement: Supplementary file 1 — Supplementary Information. [file 41598_2022_22362_MOESM1_ESM.pdf]

A pilot study of microRNA assessment as a means to identify novel biomarkers of spontaneous osteoarthritis in dogs

Atsushi Yamazaki[1], **Yuma Tomo**[1], **Hinano Eto**[1], **Koji Tanegashima**[1], **Kazuya Edamura**[1*]

[1]Laboratory of Veterinary Surgery, Department of Veterinary Medicine, College of Bioresource and Sciences, Nihon University, Fujisawa, Kanagawa, Japan

*Corresponding author
Email: edamura.kazuya@nihon-u.ac.jp

Supplementary Table 1. Raw data stats.

Sample ID	Total reads	GC (%)	Q20 (%)	Q30 (%)
UN 1	24,564,732	52.46	98.64	97.00
UN 2	24,784,647	51.63	98.69	97.11
UN 3	24,297,714	51.58	98.60	96.94
OA 1	28,609,744	53.42	98.54	96.40
OA 2	27,805,267	52.72	98.66	97.01
OA 3	26,931,737	54.48	98.47	96.54

Total reads, Total number of reads; Q20 (%), Ratio of bases that have phred quality score greater than or equal to 20; Q30 (%), Ratio of bases that have phred quality score greater than or equal to 30.

Supplementary Table 2. Summary of read preprocessing.

Sample ID	Total read count	Remain read count	Filtered read count
UN 1	19,681,778	19,096,310 (97.03%)	585,468 (2.97%)
UN 2	23,016,958	22,955,192 (99.73%)	61,766 (0.27%)
UN 3	21,675,113	21,263,578 (98.1%)	411,535 (1.9%)
OA 1	17,460,794	16,601,130 (95.08%)	859,664 (4.92%)
OA 2	18,219,055	17,523,581 (96.18%)	695,474 (3.82%)
OA 3	15,594,422	15,232,912 (97.68%)	361,510 (2.32%)

Total read count, Total read count of each sample; Remain read count, Remaining reads count after removing rRNA; Filtered read count, Filter out read count after removing rRNA.

Supplementary Table 3. Primer list for RT-qPCR.

Primer name	Official symbol	Catalog number
Cf_miR-127_1	miR-127	MS00029435
Cf_miR-542_1	miR-542	MS00030912
Cf_miR-369_1	miR-369	MS00030520
Cf_miR-381_1	miR-381	MS00030604
Cf_miR-543_1	miR-543	MS00030919
Cf_miR-146a_1	miR-146a	MS00029617
Cf_miR-338_1	miR-338	MS00030429
Cf_miR-208b_1	miR-208b	MS00030030
Hs_miR-1249_1	miR-1249	MS00014245
Cf_miR-145_1	miR-145	MS00029610
Cf_miR-144_1	miR-144	MS00029603
Cf_miR-29a_1	miR-29a	MS00030240
Hs_RNU6-2_1	RNU6-2	MS00033740
Cf_let-7a_1	let-7a	MS00029288
